# SMG-1 serves as a prognostic indicator for the radiotherapy response in head and neck squamous cell carcinoma xenografts and patients: SMG-1 serves as a radiotherapy response indicator for HNSCC

**DOI:** 10.3724/abbs.2024180

**Published:** 2024-11-04

**Authors:** Xiaofeng Wang, Yuxia Zou, Ren-Bo Ding, Xueying Lyu, Yuanfeng Fu, Xuejun Zhou, Zhihua Sun, Jiaolin Bao

**Affiliations:** 1 Department of Otolaryngology-Head and Neck Surgery the First Affiliated Hospital of Hainan Medical University Haikou 571199 China; 2 Key Laboratory of Tropical Biological Resources of Ministry of Education School of Pharmaceutical Sciences Hainan University Haikou 570228 China; 3 Cancer Centre Faculty of Health Sciences University of Macau Macao 999078 China; 4 College of Animal Science and Technology Shihezi University Shihezi 832003 China

Head and neck cancer (HNC) is one of the most common types of malignant cancer worldwide. These cancers originate in the oral cavity, oropharynx, larynx, hypopharynx, nasopharynx and sinonasal tract. The most common HNC type is head and neck squamous cell carcinoma (HNSCC). In most cases, radiotherapy serves as a primary treatment regimen for HNSCC. Therefore, investigating prognostic indicators for the response of HNSCC patients to radiotherapy is particularly important and necessary for meeting clinical needs.

Suppressor with morphogenetic effects on genitalia family number 1 (SMG-1) belongs to the family of phosphoinositide 3-kinase (PI3K)-related kinase (PIKK) family and was initially found to function as a critical component of nonsense-mediated mRNA decay (NMD), an evolutionarily conserved RNA surveillance mechanism during which transcripts containing premature termination codons are eliminated to prevent the accumulation of truncated proteins [1]. SMG-1 has also been implicated in NMD-independent functions, such as G1/S checkpoint regulation of the cell cycle, the DNA damage response, and nonhomologous end joining (NHEJ) [ 1, 2]. *SMG-1* is considered a cancer-associated gene for multiple tumors; however, its roles are still controversial among different cancer types. The upregulation or overexpression of SMG-1 potentially suppresses tumor growth and metastasis, whereas SMG-1 downregulation promotes tumor progression in gastric cancer and hepatocellular carcinoma [ 1 , 3, 4]. In these studies, SMG-1 was shown to exhibit tumor suppressive properties. However, contradictory findings have also been reported by other studies, suggesting diverse roles of SMG-1 in cancer. *SMG-1* knockdown inhibited pancreatic cancer cell proliferation and increased chemosensitivity [5]. The downregulation of SMG-1 by promoter hypermethylation was correlated with improved survival in HPV-positive HNSCC patients [6]. These studies suggest the oncogenic functions of SMG-1. Thus far, the exact function by which SMG-1 participates in human carcinogenesis and treatment response remain unclear and might be cancer type dependent. In HNSCC, further investigations to clarify the characteristics of SMG-1 with reliable evidence from animal experiments and clinical data are urgently needed.

Previous studies have demonstrated that SMG-1 has diverse expression patterns among different cancer types [ 3– 5, 7– 9]. To explore tumor-specific SMG-1 expression pattern among more cancers, we compared SMG-1 mRNA levels between tumor and adjacent normal tissues across 31 cancer types ( Figure 1A). Interestingly, we detected higher SMG-1 expression in several cancers, including HNSCC, than in normal tissues ( Figure 1B). We further analyzed an additional dataset of HNSCC patients and obtained consistent results ( Figure 1C).

**Figure 1:**
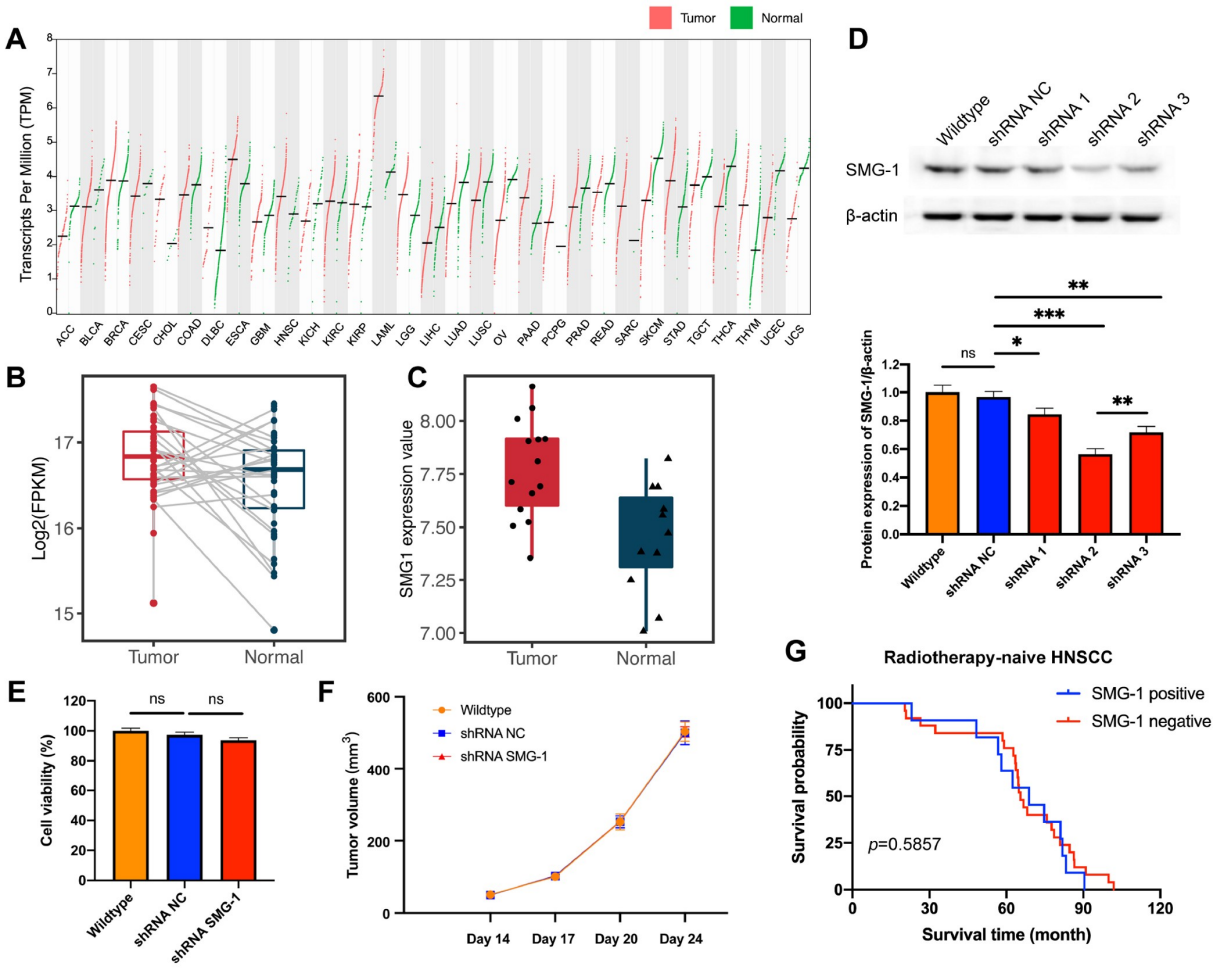
Figure 1 SMG-1 was expressed at higher levels in HNSCC tissues than in normal tissues but had no significant effect on HNSCC tumor growth or patient prognosis (A) Comparison of SMG-1 expression between tumor and normal tissues across 31 different cancer types. Diverse expression patterns were observed. (B,C) Comparison of SMG-1 expressions between HNSCC and normal tissues. SMG-1 was expressed at higher levels in HNSCC tissues than in normal tissues (P=0.019 and 0.003). Data were obtained from the TCGA database (A,B) and GEO database under access number GSE143224 (C). (D) Validation of the SMG-1 knockdown effect by western blot analysis. (E) Comparison of cell viability among wild-type, shRNA NC-transfected and shRNA SMG-1-transfected SCC-090 cells. (F) Tumor growth curves demonstrated that SMG-1 knockdown did not affect HNSCC tumor growth in SCC-090 mouse xenografts (P>0.05). (G) Kaplan-Meier analysis revealed that there was no significant difference in survival time between SMG-1-positive (n=11) and SMG-1-negative ( n=25) patients (median overall survival: 68.83 months vs 65.50 months, P>0.05). Statistical significance was calculated via two-sided t tests. *P<0.05, **P<0.01, *** P<0.001, and ns indicates P>0.05.

To investigate whether SMG-1 serves as a potential oncogene to promote HNSCC tumor growth. We generated SMG-1-knockdown SCC-090 HNSCC cells via shRNA ( Figure 1D). We subsequently examined whether SMG-1 silencing has a growth inhibitory effect on SCC-090 cells. The MTT assay results demonstrated that SMG-1 knockdown did not affect HNSCC cell proliferation in vitro ( Figure 1E). We also validated the results in an SCC-090 mouse xenograft model and confirmed that silencing of SMG-1 had no effect on HNSCC tumor growth ( Figure 1F).

To investigate whether SMG-1 expression contributes to HNSCC patient prognosis. We collected a HNSCC cohort consisting of 36 radiotherapy-naïve patients, and all patients received standard surgical resection treatment only ( Supplementary Table S1). We used immunohistochemistry (IHC) to detect tumor SMG-1 protein expression in these patients and divided all patients into two groups accordingly: SMG-1 positive ( *n*=11) and SMG-1 negative ( *n*=25). Kaplan-Meier analysis revealed that the median overall survival (68.83 months vs 65.50 months, *P*=0.5857), 3-year overall survival (90.91% vs 84.00%, *P*=0.5908) and 5-year overall survival (63.64% vs 76.00%, *P*=0.4455) rates were not significantly different between two groups ( Figure 1G and Supplementary Table S1). Taken together, these results suggest that SMG-1 modulation and expression status have no effect on HNSCC tumor growth or prognosis.

SMG-1 was previously reported to be associated with the DNA damage response to IR *in vitro* [ 6 , 10]. In the present study, we further investigated the potential impact of SMG-1 on HNSCC radiosensitivity. SCC-090 HNSCC cells transfected with shRNA NC or shRNA SMG-1 were treated with different doses of IR (0, 2, 4, 6 and 8 Gy) for various durations (24, 48 and 72 h). The MTT results demonstrated that SMG-1 silencing significantly increased the radiosensitivity of SCC-090 cells, especially for longer periods (48 and 72 h), after high-dose IR treatment (8 and 10 Gy) ( Figure 2A). We further validated these results with colony formation experiments and found that SMG-1-knockdown SCC-090 cells formed remarkably fewer colonies than the control cells under IR treatment ( Figure 2B and Supplementary Figure S1). These results demonstrated that silencing of SMG-1 contributed to enhanced radiosensitivity in HNSCC *in vitro*.

**Figure 2:**
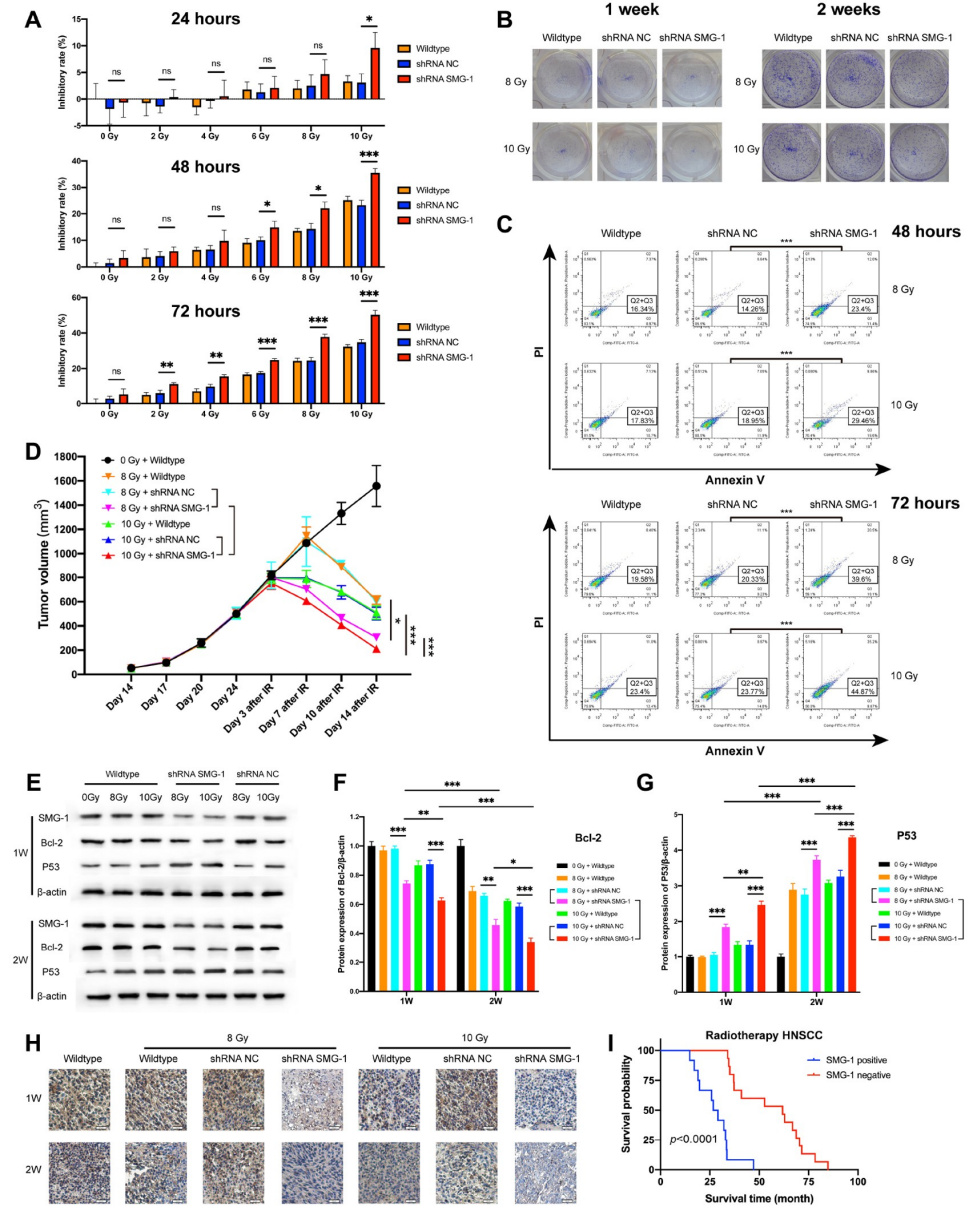
Figure 2 SMG-1 inhibition sensitized HNSCC to radiotherapy (A) Comparison of growth inhibition rates among wild-type, shRNA NC-transfected and shRNA SMG-1-transfected SCC-090 cells treated with 0, 2, 4, 6, 8 and 10 Gy IR for 24, 48 and 72 h. (B) Colony formation assay was performed to compare the SCC-090 cell proliferation rates among the wild-type, shRNA NC and shRNA SMG-1 groups treated with 8 and 10 Gy IR for 1 week and 2 weeks. (C) SMG-1 inhibition increased the degree of apoptosis induced by IR in HNSCC. Flow cytometry measurements of apoptosis in SCC-090 cells treated with 8 Gy and 10 Gy IR for 48 h and 72 h. (D) Tumor growth curves demonstrated that SMG-1 knockdown increased radiosensitivity in SCC-090 mouse xenografts after 8 and 10 Gy IR treatment for 2 weeks. (E) Western blot analysis of SMG-1, Bcl-2 and P53 protein expressions in IR-treated SCC-090 mouse tumor tissues transfected with shRNA NC or shRNA SMG-1. Quantitative analysis of Bcl-2 (F) and P53 (G) protein expressions in the wild-type, shRNA NC and shRNA SMG-1 groups. SMG-1 inhibition significantly reduced Bcl-2 expression and increased P53 expression under radiotherapy conditions. (H) Representative IHC images of Bcl-2 protein expression in tumor tissues from the wild-type, shRNA NC and shRNA SMG-1 groups. SMG-1 inhibition consistently reduced Bcl-2 expression under IR treatment. Scale bar: 20 μm. (I) Kaplan-Meier analysis demonstrated the survival time of SMG-1-positive (n=12) and SMG-1-negative (n=15) HNSCC patients who received radiotherapy. The SMG-1-negative group exhibited significantly better overall survival than the SMG-1-positive group (median overall survival: 61.83 months vs 27.92 months, P<0.0001). Statistical significance was calculated via two-sided t tests. *P<0.05, **P<0.01, ***P<0.001, and ns indicates P>0.05.

Radiotherapy can reduce tumor growth by inducing apoptosis. To explore how SMG-1 silencing enhances radiosensitivity, we stained *SMG-1*-knockdown and control SCC-090 cells with Annexin V-FITC and PI dyes and subjected them to IR treatment. Through flow cytometry analysis, we found that IR led to remarkable apoptosis in control SCC-090 cells, while silencing of *SMG-1* significantly exacerbated the apoptosis of HNSCC cells ( Figure 2C and Supplementary Figure S2).

To further confirm the effect of SMG-1 silencing, we performed a mouse xenograft experiment for validation in vivo. SCC-090 cells transfected with shRNA SMG-1 or shRNA NC were inoculated into nude mice. Compared with parental wild-type tumors and control shRNA NC tumors, shRNA SMG-1 tumors presented significantly lower tumor volumes under IR treatment ( Figure 2D). This result indicated that silencing of SMG-1 could sensitize HNSCC to radiotherapy in HNSCC.

To investigate the underlying mechanism by which SMG-1 regulates radiosensitivity in HNSCC, we detected dose-dependent and time-dependent changes in the protein expressions of Bcl-2 and P53. Bcl-2 and P53 are essential proteins involved in antiapoptotic and DNA damage responses. The western blot analysis results revealed that SMG-1 knockdown significantly decreased Bcl-2 expression and increased P53 expression under radiotherapy conditions ( Figure 2E‒G). The decreased expression of Bcl-2 and increased expression of P53 were exacerbated by higher IR doses and longer IR exposure times ( Figure 2E‒G), which were associated with greater tumor growth inhibition ( Figure 2D). Consistent findings were also demonstrated by the IHC staining results ( Figure 2H and Supplementary Figure S3). These data suggest that silencing of SMG-1 enhances radiosensitivity in HNSCC, likely through the modulation of Bcl2 and P53-mediated antiapoptotic effects.

On the basis of the findings that SMG-1 is strongly associated with radiosensitivity in vitro and in vivo in HNSCC, we hypothesized that low expression of SMG-1 could serve as a prognostic indicator for the response of HNSCC patients to radiotherapy. We collected a HNSCC cohort consisting of 27 patients who received radiotherapy ( Supplementary Table S2). All HNSCC patients were divided into two groups according to SMG-1 protein expression, including 12 SMG-1-positive patients and 15 SMG-1-negative patients. Compared with the SMG-1-positive group, the SMG-1-negative group presented significantly better median overall survival (61.83 months vs 27.92 months, *P*<0.0001), 3-year overall survival rates (80.00% vs 8.33%, *P*=0.0002) and 5-year overall survival rates (53.33% vs 0.00%, *P*=0.006) ( Figure 2I and Supplementary Table S2). Taken together, these results suggest that SMG-1 could serve as a prognostic indicator for the response of HNSCC patients to radiotherapy.

In the present study, we investigated the potential prognostic contribution of SMG-1 to HNSCC in multiple aspects, including *in vitro* and *in vivo* experiments ( Supplementary Methods), to examine the impact of *SMG-1* silencing on HNSCC cell growth and the response to radiotherapy and retrospectively analyzed the survival of SMG-1-positive and SMG-1-negative HNSCC patients with and without radiotherapy. Our results revealed several notable findings, including the following: (1) SMG-1 exhibited higher expression levels in HNSCC tissues than in normal tissues but had no significant effects on HNSCC tumor growth; (2) silencing of *SMG-1* sensitized HNSCC tissues to radiotherapy, and lower SMG-1 expression in HNSCC patients contributed to better survival time in patients receiving radiotherapy. These results revealed the cancer-regulating role of SMG-1 as a negative radiosensitivity mediator; (3) SMG-1 could serve as a prognostic indicator for the response of HNSCC patients to radiotherapy, which provides a new opportunity to guide precision medicine for HNSCC patients.

## Supplementary Data

Supplementary data is available at *Acta Biochimica et Biphysica Sinica* online.
